# Elevation-Dependent Temperature Trends in the Rocky Mountain Front Range: Changes over a 56- and 20-Year Record

**DOI:** 10.1371/journal.pone.0044370

**Published:** 2012-09-06

**Authors:** Chris R. McGuire, César R. Nufio, M. Deane Bowers, Robert P. Guralnick

**Affiliations:** 1 University of Colorado Natural History Museum, University of Colorado, Boulder, Colorado, United States of America; 2 Environmental Studies Program, University of Colorado, Boulder, Colorado, United States of America; 3 Department of Ecology and Evolutionary Biology, University of Colorado, Boulder, Colorado, United States of America; University of Oxford, United Kingdom

## Abstract

Determining the magnitude of climate change patterns across elevational gradients is essential for an improved understanding of broader climate change patterns and for predicting hydrologic and ecosystem changes. We present temperature trends from five long-term weather stations along a 2077-meter elevational transect in the Rocky Mountain Front Range of Colorado, USA. These trends were measured over two time periods: a full 56-year record (1953–2008) and a shorter 20-year (1989–2008) record representing a period of widely reported accelerating change. The rate of change of biological indicators, season length and accumulated growing-degree days, were also measured over the 56 and 20-year records. Finally, we compared how well interpolated Parameter-elevation Regression on Independent Slopes Model (PRISM) datasets match the quality controlled and weather data from each station. Our results show that warming signals were strongest at mid-elevations over both temporal scales. Over the 56-year record, most sites show warming occurring largely through increases in maximum temperatures, while the 20-year record documents warming associated with increases in maximum temperatures at lower elevations and increases in minimum temperatures at higher elevations. Recent decades have also shown a shift from warming during springtime to warming in July and November. Warming along the gradient has contributed to increases in growing-degree days, although to differing degrees, over both temporal scales. However, the length of the growing season has remained unchanged. Finally, the actual and the PRISM interpolated yearly rates rarely showed strong correlations and suggest different warming and cooling trends at most sites. Interpretation of climate trends and their seasonal biases in the Rocky Mountain Front Range are dependent on both elevation and the temporal scale of analysis. Given mismatches between interpolated data and the directly measured station data, we caution against an over-reliance on interpolation methods for documenting local patterns of climatic change.

## Introduction

Mountainous regions, especially the alpine, are among the environments most vulnerable to the effects of climate change [1]–[3]. Climatic changes in mountains have had and will likely continue to have strong impacts on hydrologic cycles [4], [5], the timing of biological events [6]–[8], and biodiversity [9], [10]. For biological systems adapted to narrowly distributed and extreme environments, even small changes in climatic conditions can have proportionally large impacts on flora and fauna [11]. An essential first step toward predicting hydrologic and ecosystem changes in individual mountain systems is to document climatic trends at various elevations within these systems.

Despite much work documenting temperature trends across mountain systems of the world over the last half century, a consistent elevation-dependent or systematic climate change signal has not become apparent [12]–[14]. For example, while warming trends in the European Alps [15], [16] and the Tibetan Plateau [17] strengthen with elevation, warming trends in the tropical Andes have been found to weaken with elevation [18]. Given these differences, the key question is whether generalities that can account for this variability will emerge as further case studies in different regions of the world become available. Further, the variability in elevation-dependent climatic trends points to the need to develop climate monitoring along mountain transects that can control for regional heterogeneity and latitudinal influence while improving spatial resolution.

In North America, the only known long-term climate transect representative of a continental mountain range is located in the Rocky Mountain Front Range immediately west of Boulder, Colorado. Situated along the 40th parallel, the Rocky Mountain Front Range transect (hereafter the RMFR transect) runs from 1672 m in the high plains to 3749 m in the alpine tundra over a distance of less than 30 km. The base of this transect in Boulder, Colorado, has been collecting temperature data over the last 100 years and is currently serviced by the United States Department of Commerce’s National Oceanic and Atmospheric Administration (NOAA). The four mountain stations west of Boulder have been collecting daily temperature data in the Colorado Front Range since 1952 [19] and are serviced as part of the Niwot Ridge Long-Term Ecological Research (NWT LTER) project of the University of Colorado in Boulder.

Studies modeling the effects of climate change within the Rocky Mountain region generally predict that warming trends will be greatest at higher elevations, with the alpine showing the greatest relative change [20]–[22]. In contrast, studies incorporating hydrological changes near the Front Range of Colorado predict a mid- to high-elevation cooling [23]. Both sets of studies contrast with previous examinations of the climate record of the RMFR transect (1952–1998), which found warming associated with stations below 3000 m, and, counter to expectation, that the alpine was associated with significant cooling [24], [25]. These studies suggest that climate change across a vast area such as the Rocky Mountains may be more complex than expected. However, a more detailed understanding of this complexity is precluded by the lack of a complete contemporary analysis of the temperature trends, such as that provided by the RMFR transect. The RMFR transect contains the best non-interpolated and latitudinally controlled data available for the Rocky Mountain Front Range and, therefore, its analysis and interpretation are essential for improving the spatial resolution of trends in the Rocky Mountains. The accelerated pace of Northern Hemisphere warming in recent decades [26] further necessitates a thorough examination of the temperature record associated with the RMFR transect.

The purpose of this study is to provide an analysis and comparison of temperature trends along the 2077 m elevational gradient of the RMFR transect over both a 56-year (1953–2008) and a 20-year (1989–2008) period. We describe trends over the full available record (56 years; 1953–2008) and over the last 20 years (1989–2008) in order to compare long-term (>50 years) temperature trends to temperature trends during the recent period of accelerating global climatic change [26]. The data compared include trends in average yearly and monthly maximum, minimum and mean temperatures. Monthly temperature trends on both temporal scales were examined to determine the finer-scale seasonal basis for detected changes.

As interpolation of climate data within mountain systems can be difficult given complex topologies and the low density of available weather stations [27], [28], access to the 56-year RMFR data allowed us to test the ability of the Parameter-elevation Regression on Independent Slopes Model (PRISM; http://www.prism.oregonstate.edu/) to accurately interpolate the yearly temperature values (max, min, mean) and their estimated rate of climate change over the last 56-years at each site along the RMFR transect. Low correlations between the interpolated and actual data sets and significant differences in their measured rates of climate change over the last 56-years would highlight the value of long-term data sets and provide caution about overreliance on interpolated data for understanding climate change along gradients, especially in the Rocky Mountains where a variety of different patterns of future climate change have been proposed [22], [23]. Finally, as there is great interest in how climate change is affecting biological indicators (e.g. [6]–[8]), we also explored how changing temperatures have influenced growing season length and available growing degree days (GDD) along the elevational transect.

## Methods

### Rocky Mountain Front Range Transect

**Figure 1:**
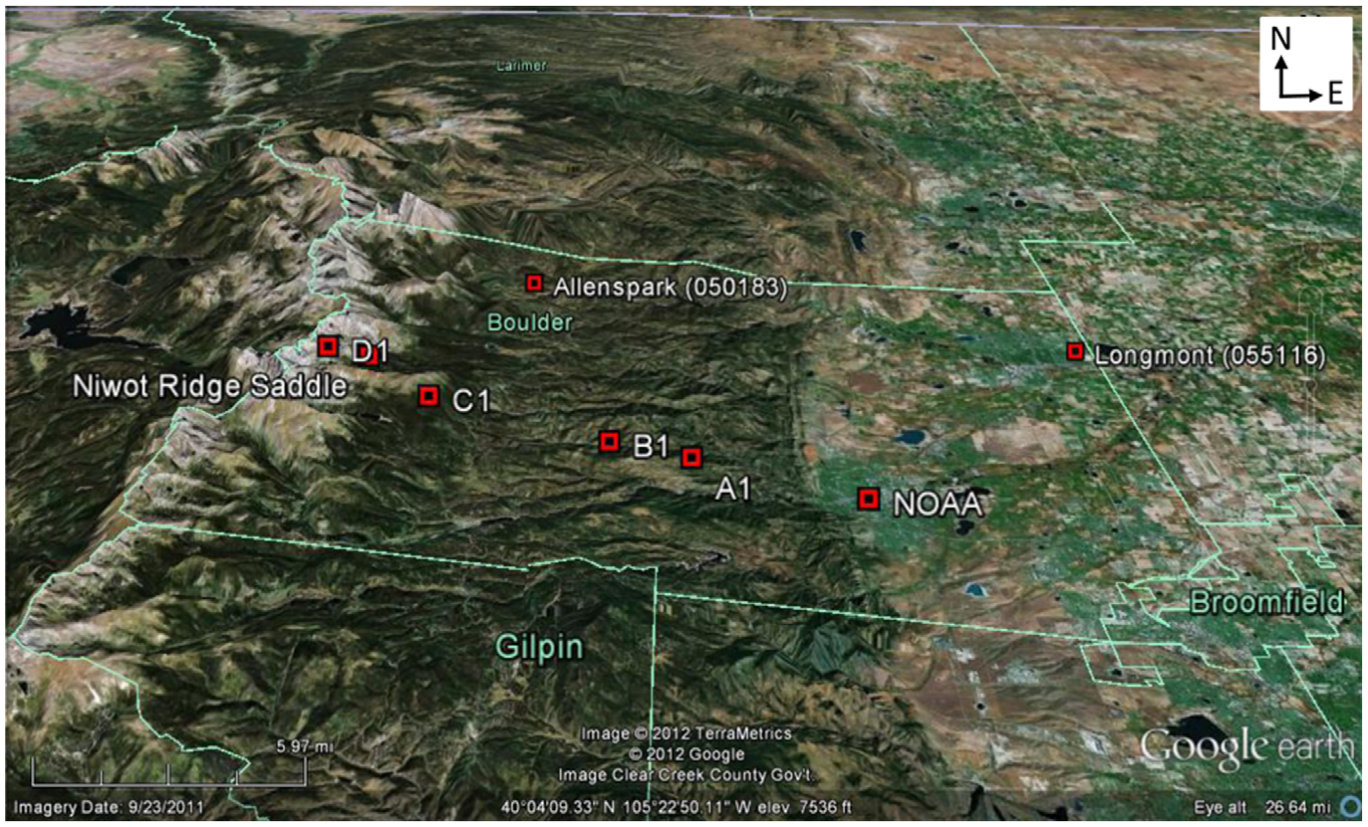
Topographic relief map showing locations of RMFR transect and important stations used for infilling and data correction (Denver station not shown).

Four of the five stations that make up the RMFR transect were established in 1952 by John Marr of the University of Colorado [19], [29], [30]. Data for these stations were provided by the Niwot Ridge Long-Term Ecological Research (NWT LTER) project and the University of Colorado Mountain Research Station. The goal for establishing these weather stations was to provide detailed climatic information on distinct ecological zones of the Eastern slope of the Rocky Mountain Front Range. The stations are referred to as A1 (2185 m), B1 (2591 m), C1 (3048 m), and D1 (3749 m), and they reflect climate associated with a regional lower montane (foothills), an upper montane, a subalpine and an alpine tundra life zone, respectively (Table 1; Figure 1). To minimize potential biases associated with the placement of these weather stations and to assure that they reflected climate associated with the ecological zones they were to represent, Marr placed these stations in areas with soils and topographic settings that were moderate for their type, that were near the center of each designated life zone, and that were along a single ridge system that followed the 40^th^ parallel [31]. Because we could not pair other local weather stations for similarity in topography, instrumentation used, temporal scale of data collection and latitude, data from other stations were used to help correct anomalous data events but, as has been done in previous studies [24], they were not included in our measures of local climate change. The RMFR transect expands Marr’s original transect by including a fifth weather station currently located at the National Institute of Standards and Technology (NIST) building on the western edge of Boulder, south of the United States Department of Commerce campus in Boulder, Colorado. This weather station is serviced by the National Oceanic and Atmospheric Administration (NOAA; 1672 m) and reflects climate associated with the local high plains-foothills ecotone. Data for NOAA (Cooperative ID 050848) were obtained from the U.S. Department of Commerce National Climatic Data Center.

**Table 1:** Weather stations along the Rocky Mountain Front Range Climate Transect and additional stations used to correct anomalous readings and infill missing climate data.

Site	Elevation(m)	Ecological zone Classification	Latitude	Longitude
NOAA	1672	High plains	39.992	−105.267
A1	2185	Lower montane	40.015	−105.377
B1	2591	Upper montane	40.023	−105.430
C1	3048	Subalpine	40.036	−105.547
D1	3749	Alpine tundra	40.059	−105.617
**Additional sites**				
Longmont (055116)	1508	High plains	40.158	−105.073
Denver (052220)	1611	High plains	30.763	−104.869
Niwot Ridge Saddle	3525	Alpine tundra	40.054	−105.589
Allenspark (050183)	2504	Upper montane	40.188	−105.501

Daily minimum and maximum temperatures have been recorded at stations A1, B1, C1, and D1 since 1953. When necessary, these data were normalized and adjusted for instrument change (see [24]). In addition, the replacement of the hygrothermographs by electronic datapods at A1 and B1 in 1987 resulted in a need to set the lower temperature limits of the total max and min records (1953–2008) for these two sites at −17.8°C because the datapods could not record temperatures below this threshold. Daily minimum and maximum temperatures for Boulder, Colorado, have been continuously recorded since 1897, excepting a period from 1989 to 1990. From 1947 to 1989 temperature data were recorded by the Boulder Fire Department from instrumentation located on the fire station grounds. From 1990 onwards, weather observations from Boulder were collected from the NIST building and administered by NOAA. A previous analysis of the RMFR transect data [24] included an analysis of observations from Longmont, Colorado (Cooperative ID 055116). Our study does not include an analysis of the Longmont data due to the station’s inactivity since 2004. However, the stability of the Longmont station’s location over its historical record enables controlling for instrument location change associated with the Boulder data. Using the Longmont station as a stable guide, temperature data for Boulder for the period 1952–1989 were adjusted to reflect a stable location at NIST across the data record (methods discussed further below).

As noted and detailed by other studies, several gaps are present within the temperature records of the RMFR transect stations [24], [32]. Infilling these gaps, which ranged from days to (on rare occasions) months, was necessary to identify frost dates and calculate seasonal GDDs. In addition to infilling gaps, we further screened the individual climate records for anomalous temperature values. The protocol for infilling gaps and correcting anomalous values is outlined below. As weather data for sites A1 and B1 between the years 1970 and 1986 contained large multi-month gaps (with adequate weather data for only 59 out of 204 months at A1 and for only 88 of 204 months at B1), and because there are no data available for NOAA for several months across 1989 and 1990, these years were excluded from all analyses of the respective stations. The large gaps across multiple years at A1 and B1 were not infilled to avoid an over reliance on non-independent data that could mask potential differences in climate trends at each elevation. While the exclusion of the 1970–1986 data from NOAA, C1 and D1 did not affect the interpretation of their temperature trends in any appreciable manner (see below), these years were included at these sites to increase statistical power and decrease the probability of a type II error. All uncorrected, corrected and infilled RMFR weather data used in this study and the description of methods used to correct or infill data are available as Appendix S1.

As discussed below and summarized in Tables 1 and 2, temperature data from adjacent climate stations external to the RMFR transect were used during the data correction process. Data for the Niwot Ridge Saddle (3525 m) were provided by the NWT LTER project and the University of Colorado Mountain Research Station. Data for Longmont, Colorado (Cooperative ID 055116), Denver, Colorado (Cooperative ID 052220) and Allenspark, Colorado (Cooperative ID 050183) were obtained from the U.S. Department of Commerce’s National Climate Data Center.

**Table 2:** Hierarchy of sites used to infill and correct anomalous data at each weather station.

Site	Hierarchy of sites used fordata corrections	Total % infilledMax Min	# of events where infilled data was equal to 7–13 daysMax, Min	# of events where infilled data was >than 13 days Max, Min	% anomalousMax Min
NOAA	Longmont, Denver, Allenspark	1.1% 1.2%	0, 0	6, 6	1.9% 1.3%
A1	B1, NOAA, C1, Allenspark	3.3% 3.3%	3, 3	9, 9	1.5% 0.7%
B1	A1, C1, NOAA, Allenspark	3.0% 3.0%	5, 5	7, 7	0.9% 1.3%
C1	Saddle, B1, D1, Allenspark	2.5% 2.5%	15,11	7, 8	0.2% 0.7%
D1	C1	6.2% 6.2%	39, 47	14, 16	0% 0%

Information provided on the number of times infilled data exceeded a week or two of consecutive days across the complete data record at each site. See text and Appendix S1 for details.

### Gap Filling and Data Corrections

Gaps in the temperature record from C1 and D1 were originally filled by the NWT LTER data manager using linear regression methods developed by Greenland [32]. Briefly, the 14 days before and after the data gaps were regressed on the corresponding values from adjacent weather stations. The resulting regression equation was then used to interpolate missing temperature values for the focal station. The detailed methodology and the hierarchy of adjacent stations used for this process are outlined on the NWT LTER website (http://culter.colorado.edu/exec/nwtdatas.cgi). This infilling method was used only to infill data for C1 and D1 when the regression r^2^ value exceeded 0.6. Due to missing data for reference stations, data for five dates at C1 could not be infilled using this method and instead were infilled using the average of the temperatures for the date before and the date after the missing data point (see Appendix S1).

An additional level of data infilling was completed in preparation for analyses in this manuscript. This is because there remained gaps in the data records for A1, B1, and NOAA that had not previously been infilled by the NWT LTER data managers, and several gaps remained at stations C1 and D1 due to low correlations between the temperatures of these stations and adjacent stations during the 14 days prior to and following a particular gap. To address low station correlations and to add ease and speed to the infilling process, we developed a regression-by-month infilling method. The temperature relationship between any two adjacent sites is not constant across the year but, rather, it varies seasonally due to synoptic (such as wind flow direction and thermal inversion) and orographic conditions as well as host of other variables. Accordingly, the use of 14-day post and prior windows that spill over into multiple months may promote low regression r^2^ values which diminish predictive power as the size of a gap increases. A regression that is confined to a single month but incorporates several years avoids this limitation while offering a much larger pool of data to determine the statistical relationship of temperatures between sites during the month of interest. Further, whereas a ±14-day window requires a separate regression calculation for every gap, the regression-by-month method results in only 12 regression formulae (one for each month at each site) that are used to infill gaps that fall within a given month).

The regression-by-month methodology is as follows: the available temperature values from the focal station for each date within a calendar month across the entire data record were regressed onto the corresponding values from an adjacent weather station. Using the resulting regression formula for a given month, known temperature values from the adjacent station were used to predict and infill missing temperature data for the focal station. The hierarchy of adjacent predictor stations used for each focal station is shown in Table 2. This hierarchy follows the general pattern of nearest to farthest in elevation and distance to the focal station and, accordingly, from highest to lowest correlation with temperatures between the focal station and the predictor stations for a given month. In most cases, the highest correlation was with the RMFR transect stations nearest to the focal station.

In cases where gaps exceeded one month or where single site comparisons for a given month produced regression r^2^ values of less than 0.60, two adjacent predictor sites were used instead of one. The use of two predictor sites compensated for differences in the relationship between ambient temperatures at two sites that may have been caused by changes in synoptic conditions (such as wind flow direction) during different parts of the year. The hierarchy of predictor stations used in these cases was the same as that used for single predictor sites (Table 2). The two resulting regression formulae were used to generate two independent values for the same missing data point. The value used to fill a missing data point was the weighted average of these two predicted values, with the weight of each being proportional to their individual regression r^2^ values.

Depending on the station, data generated for gap infilling previously by NWT LTER and within this study accounted for between one and six percent of their total data record (see Table 2 and Appendix S1). Of the 738 min and max temperature values that were infilled at NOAA, 0.04% of these values were infilled using two predictor sites, while two predictor sites were used to infill 78% of the 1347 infilled temperature values at A1 and 81% of the 1245 infilled values at B1. All other data at these sites were infilled using a single predictor site. All infilled temperature values at C1 and D1, which were 1024 and 2546 temperature values respectively, only used a single predictor site.

While infilling could conceivably influence the results of trend analyses or create issues of non-independence, the inclusion of this model-generated data was important for measuring changes in yearly accumulated GDDs and growing season length. As the inclusion of this infilled data accounted for a relatively low amount of the daily temperature data associated with each weather station and as these additions were spread through months and years (Appendix S1, Table 2), this inclusion did not change the interpretation of any trend magnitudes or significance in our study when comparing against trends generated using data sets with non-filled gaps. For example, for D1, the site with the most infilled data (6.2% of min and max temperature values, Table 2), the slope of change (°C/year) over the 56-year record for max temperature was −0.005 (gaps remaining; Kendall’s Tau p = 0.745) and 0.007 (gaps filled; p = 0.276) and the slope of change for min was −0.016 (gaps remaining; p = 0.467) and −0.005 (gaps filled; p = 0.7238). In both cases the respective min and max slopes did not significantly differ whether the gaps remained or were infilled (P>0.10). Monthly and yearly averages were therefore assumed to be largely free of material non-independence and used as independent data points in temperature trend analysis.

The climate data at NOAA, A1, B1, and C1 were also screened for anomalous values. Controlling for elevation, data were considered anomalous if the median temperature difference between the focal station and its three reference stations was greater than 10°C or less than −10°C [24]. These anomalous data values were removed and the resulting gaps were infilled according to the regression-by-month methodology described above. Depending on the site, between zero and two percent of the total temperature data were found to be anomalous and were subsequently corrected (Table 2). Generally, the referenced stations for each site consisted of the RMFR transect stations immediately above and immediately below the focal station, plus a single station external to RMFR transect located in Allenspark, Colorado (Table 1). The NOAA station was referenced against three weather stations external to the RMFR transect: two stations of approximately equivalent elevation (Longmont, Colorado and Denver, Colorado) plus the station in Allenspark, Colorado. Data from D1 could not be screened for anomalous values due to the lack of appropriate reference stations at similar elevations in the Rocky Mountain Front Range.

To determine whether the change in location of the NOAA station in 1990 had an impact on the recorded temperatures, data from this site were compared against data from the stable station in Longmont, Colorado, which had recorded temperatures consistently between 1960 and 2004. The difference in average monthly temperature between NOAA and Longmont was calculated for the periods 1960–1987 and 1991–2004 and a t-test was used to determine whether these differences had changed between the two periods. We found that temperatures recorded during the months January, February, March, and April were significantly affected by the change in location (p<0.05). To correct for this, temperatures at NOAA from January to April for dates prior to 1987 were adjusted to maintain a consistent temperature difference between Longmont and Boulder across both time periods. Maximum temperatures for NOAA prior to 1987 were adjusted downward by 1.17, 0.91, 0.99 and 0.84°C for January, February, March, and April, respectively. Minimum temperatures were adjusted upward by 0.64, 0.76, 0.81, and 0.72°C for January, February, March, and April, respectively.

### Trend Analyses Over 20 and 56 Year Records

For analysis of temperature trends (i.e. the slope of change over time) over the 20 and 56-year record, average yearly and monthly mean, maximum, and minimum temperatures were regressed on year for all sites. For analyses of growing season indices, the timing of last spring frost, first autumn frost, growing season length, and annual GDDs were regressed on year for all sites. Due to potential issues of autocorrelation common to environmental time-series data, we used Kendall’s Tau nonparametric method for testing trend significance levels in all regression analyses. However, slopes measuring average changes over time were determined using linear regression models. All statistical analyses were performed using JMP 8.02 [33].

Exclusion of the missing period 1970–1986 at A1 and B1 in the analysis of the 56-year temperature trends created the potential for incommensurability between trends at these two stations and those at the remaining RMFR transect stations. To address this possibility, we compared the 56-year yearly max and min slopes from the complete data records of NOAA, C1, and D1 to the max and min slopes that resulted when the years 1970 through 1986 were removed from the data record. The slopes (°C/year) and Kendall’s Tau p-values associated with the full 56-year climate record and the data sets with 1970–1986 climate removed were 0.009 (p = 0.118) vs. 0.007 (p = 0.160) for NOAA max, −0.025 (p<0.0001) vs. −0.028 (p<0.0001) for NOAA min, and 0.045 (p<0.0001) vs. 0.042 (p<0.0001) for C1 max, −0.027 (p = 0.572) vs. −0.004 (p = 0.458) for C1 min, and finally, 0.007 (p = 0.276) vs. 0.007 (p = 0.414) for D1 max, and −0.005 (p = 0.724) vs. −0.005 (p = 0.773) for D1 min. In all cases, the max and min slopes calculated over the 56-year record for each of these sites did not differ significantly whether the 1970–1986 data was removed or retained (p>0.10). In turn, the relative significance of each trend also did not differ. That this was true at elevations higher (C1 and D1) and lower (NOAA) than stations A1 and B1 suggests that the period 1970 through 1986 may lack appreciable influence on the calculation of 56-year trends across the transect. Therefore, we felt that the absence of temperature data at A1 and B1 between 1970 and 1986 did not preclude comparison of 56-year trends at A1 and B1 to the trends of the other three stations. Finally, we choose to not reconstruct such an extensive time record for all of these years to minimize the degree of non-independence between stations.

### Interpolation of Climate Data Along the RMFR Transect

To test the ability of PRISM to interpolate climate data along the RMRF transect over last 56-years record, we compared the yearly max, min and mean temperature values of our data with the yearly temperature values interpolated by PRISM for the 2.5-minute (∼4 km) grid cell associated with the georeference coordinates of each of the RMFR weather stations (http://www.prism.oregonstate.edu/; data accessed September 2011). The interpolated yearly means were determined by averaging PRISM’s corresponding yearly max and min temperatures. A linear regression model was used to determine the degree to which the 56-yearly max, min and mean PRISM and RMFR records were correlated at each site. The interpolated yearly max, min and average values were also regressed over time to determine the estimated rates (slopes) of climate change over the last 56 years at each weather station. The PRISM slopes were then compared to the corresponding slopes associated with each of the RMFR weather stations. Differences in the rate of climate change estimated using the interpolated PRISM and actual climate data sets were compared by determining whether the 95% confidence intervals around the slopes produced by linear regression models overlapped.

### Calculation of Growing Season Indices

Growing season length is generally defined as the period between the last spring frost and the first autumn frost [34]. In our study, we calculated the last spring frost and the first autumn frost using a seven-day running average of daily minimum temperatures. The last frost leading into spring was calculated as the last date on which the seven-day running average of daily minimum temperatures transitioned to positive degrees Celsius while the first autumn frost was calculated as the first date on which the seven-day running average of daily minimum temperatures transitioned to negative degrees Celsius. A seven-day window was used in an effort to provide resistance to spurious values and to clearly demarcate transitions into and out of periods of freezing temperatures. Annual growing degree days (GDD) were calculated as Σ [(T_max_ + T_min_)/2] – T_base_ where T_max_ and T_min_ are daily maximum and minimum temperatures, respectively, and T_base_ is 10°C. Any minimum or maximum temperature below T_base_ was set to 10°C. This base temperature (10°C) is the temperature most commonly used for GDD calculations and was used in our study to allow for direct comparison with other studies [35], [36]. While there are different ways to estimate GDDs [37]–[39], the method we used is commonly employed in the climate change literature [40]–[42].

## Results

### Yearly Mean, Maximum, and Minimum Temperatures

**Figure 2:**
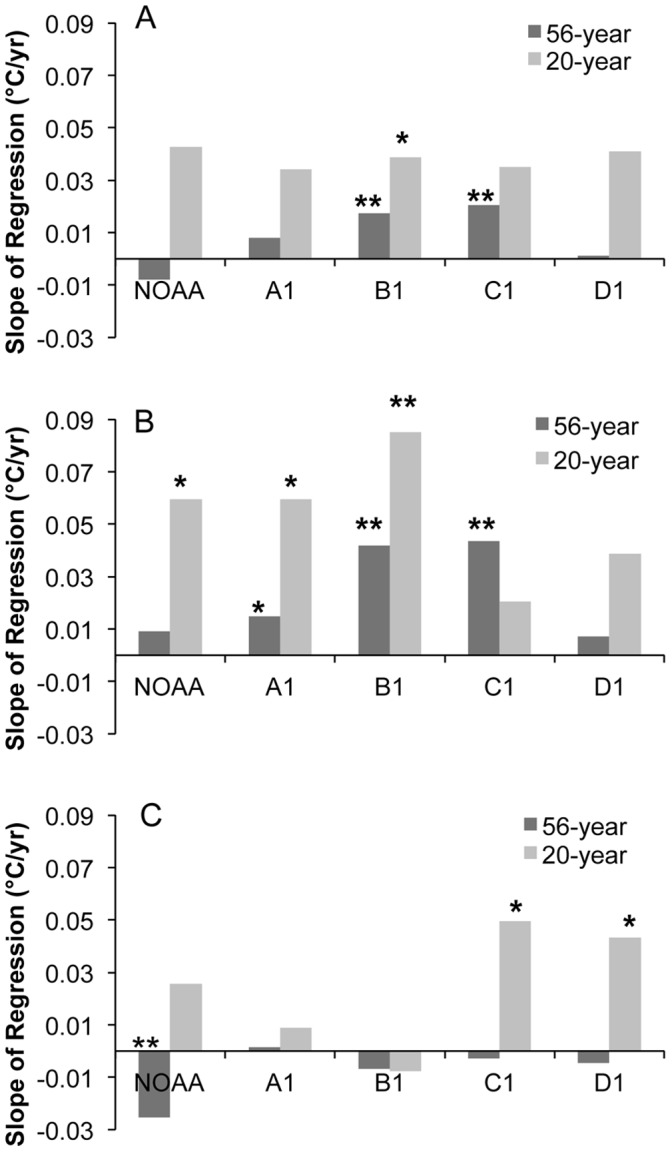
Yearly temperature trends derived by linear regressions for the periods of 1953–2008 (56-year) and 1989–2008 (20-year) across the RMFR transect. Trends shown as changes in the average yearly (A) mean, (B) maximum and (C) minimum temperatures (°C) per year. Significance level of regressions denoted by * p<0.05, ** p<0.01.

Analysis of the 56-year trends in average yearly mean temperature shows that intermediate elevations (Sites B1 and C1, 2591–3048 m) have experienced the most warming. That is, in terms of the mean temperature across the gradient, significant warming was uncovered at sites B1 (0.17°C/decade) and C1 (0.20°C/decade) while lower elevations (NOAA and A1) and the alpine (D1) showed no significant warming trends (Figure 2A). When only the recent 20-year climate record (1989–2008) is considered, however, the effect of elevation is less evident. The rate of mean warming over the last 20 years was a nearly uniform 0.4°C per decade across the transect (Figure 2A), although trends were only significant at B1 (0.39°C/decade). Non-significant warming trends at NOAA, A1, C1 and D1 were 0.43°C, 0.34°C, 0.35°C and 0.41°C/decade, respectively.

The 56-year trends in average yearly maxima show a warming trend at the three intermediate elevations (A1, B1, and C1) while both the lowest (NOAA) and the highest (D1) elevations show no significant warming (Figure 2B). The magnitude of the 56-year trends in average yearly maxima is consistent with trends of yearly means across this period, with sites B1 and C1 demonstrating the largest and most significant warming. Average yearly maximum temperatures over the 56-year record have increased at A1 at 0.15°C/decade while average maximum temperatures at B1 and C1 have increased at 0.42°C and 0.44°C/decade, respectively. Non-significant warming trends at NOAA and D1 were associated with increases of 0.09°C and 0.07°C/decade, respectively.

Over the last 20 years, significant maximum temperature increases have been confined to lower elevations (NOAA, A1, and B1) while positive slopes at C1 and D1 were not significant (Figure 2B). Average yearly maxima over the period 1989–2008 have increased at NOAA and A1 at a rate of 0.60°C/decade and at B1 at approximately 0.85°C/decade. Non-significant warming trends at C1 and D1 were associated with increases of 0.21°C and 0.39°C/decade, respectively.

The 56-year trends in average yearly minima show a significant cooling of about 0.25°C per decade at NOAA while no trends are evident at higher elevations (Figure 2C). In contrast, the 20-year trends in average yearly minima show a significant warming of 0.50°C and 0.43°C/decade at C1 and D1, respectively, while no significant trends are uncovered at lower elevations (Figure 2C).

In summary, warming over the 56 and 20-year records for all elevations below the subalpine (C1) is predominantly a function of an increase in average yearly maxima. While the 56-year record indicates that the subalpine (C1) has warmed due to increases in the maximum temperatures, the 20-year record indicates that recent warming in the alpine (D1) and subalpine (C1) has occurred through a significant increase in average yearly minimum temperatures and a noticeable but non-significant increase in yearly maxima as well. However, in contrast to increasing maximum temperatures at C1 over the 56-year record, no such warming trend exists at D1 over this period.

### Monthly Maximum and Minimum Temperatures

**Figure 3:**
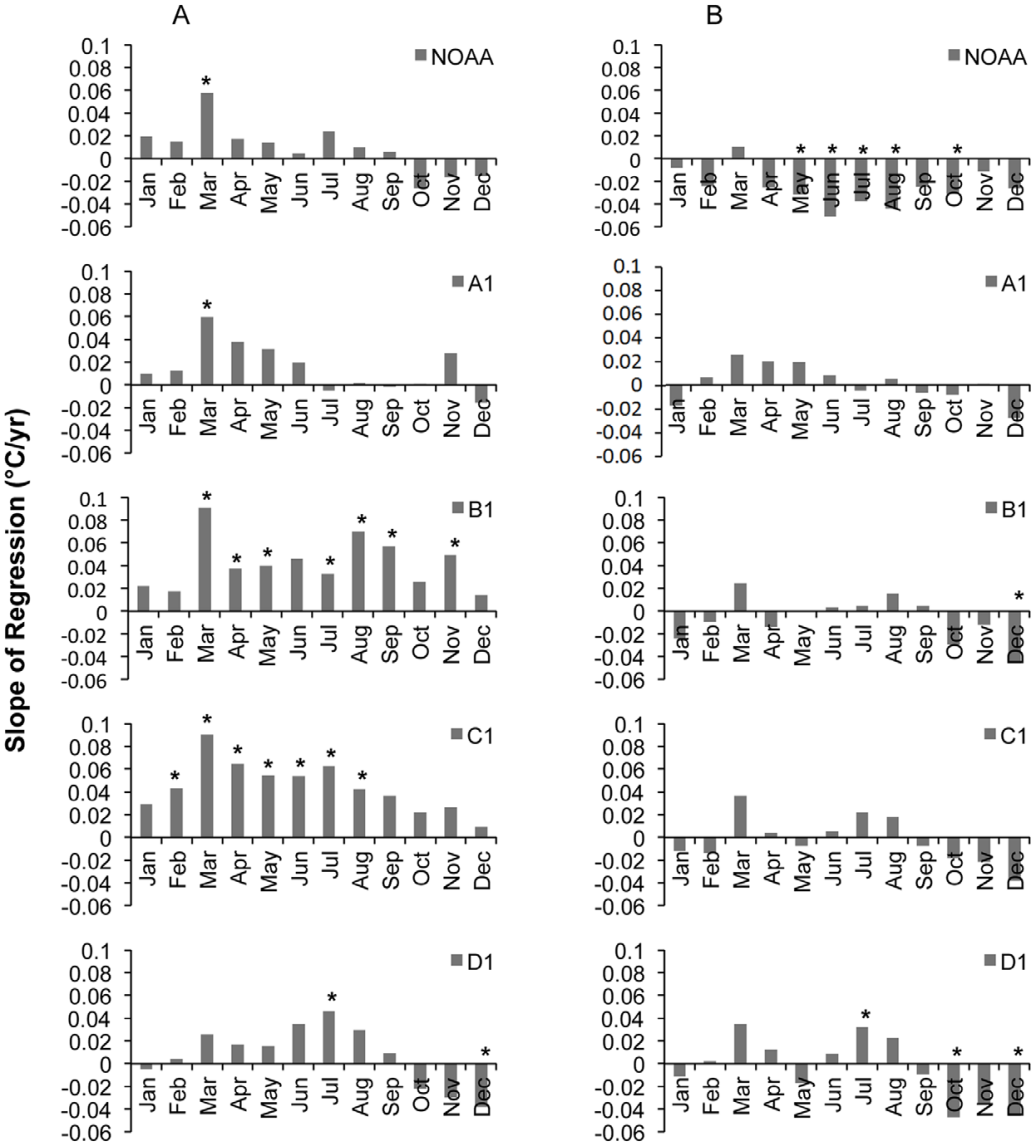
Monthly temperature trends derived by linear regressions over a fifty-six-year period (1953–2008) across the RMFR transect. Trends shown as changes in the average monthly (A) maximum and (B) minimum temperatures (°C) per year. Significance level of regression denoted by * p<0.05.

Over the past 56 years, there has been a tendency toward increases in average monthly maxima in March across all elevations below the alpine (D1), and in July and August at sites B1 and above (Figure 3A). In general, the warming trends appear to be greatest at B1 and C1. Additionally, B1 and C1 demonstrate significant increases in maxima across much of the year from February through September. At D1, there is evidence for autumn and early winter cooling, although only December produced a significant negative trend in monthly maximum temperatures (Figure 3A). Trends in average monthly minimum temperatures are largely non-significant over the 56-year record excepting the presence of a significant cooling trend from May through October at NOAA, significant warming at D1 during July, and cooling during December at B1 and D1 (Figure 3B).

**Figure 4:**
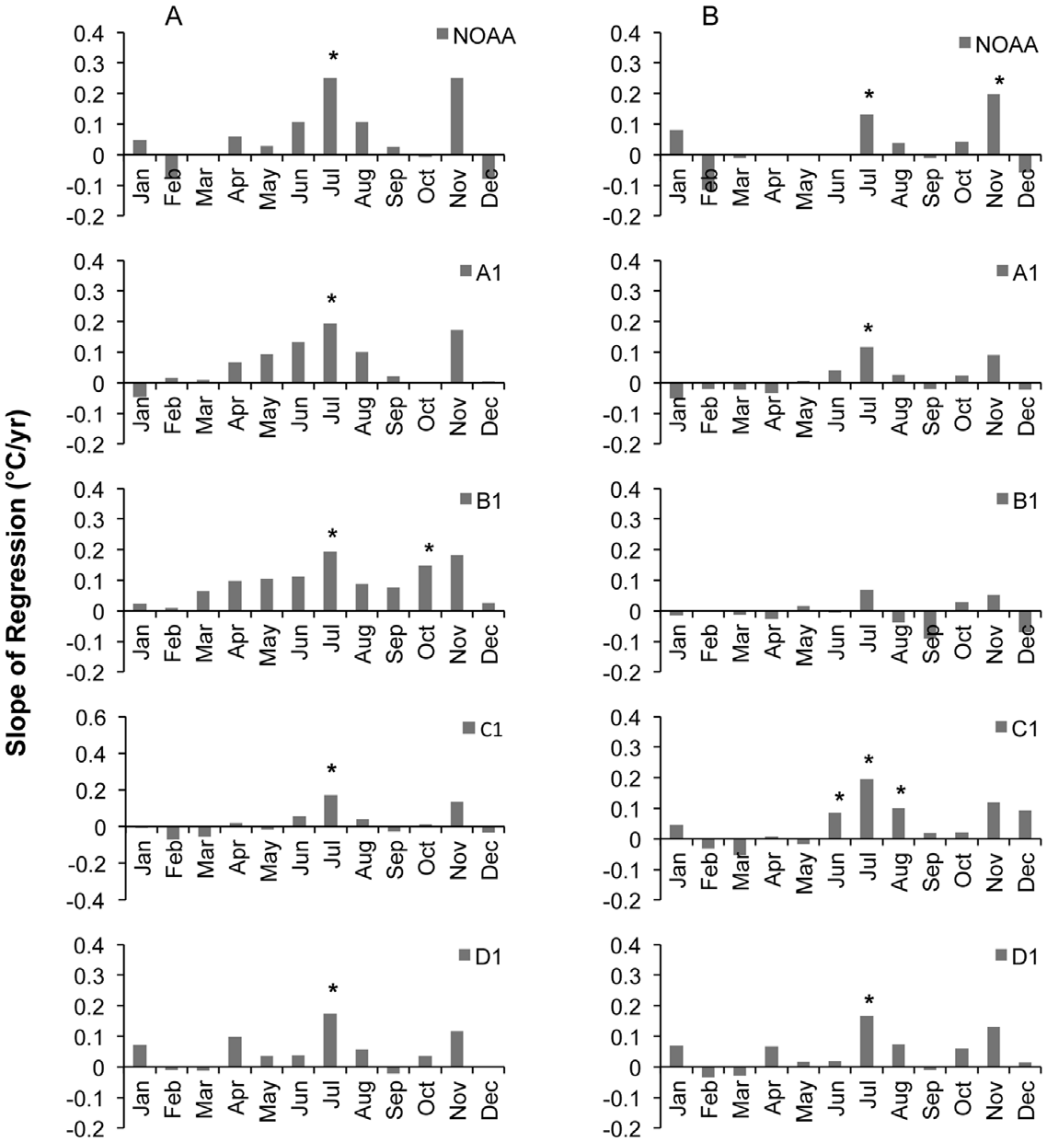
Monthly temperature trends derived by linear regressions over a twenty-year period (1989–2008) across the RMFR transect. Trends shown as changes in the average monthly (A) maximum and (B) minimum temperatures (°C) per year. Significance level of regression denoted by * p<0.05.

Over the 20-year record, the magnitude and direction of monthly maximum and minimum temperature trends are highly consistent across the RMFR transect. The strongest warming signals occur in July and November for both the maximum and minimum temperatures (Figure 4AB), although trends in November are generally non-significant. On average, the fairly uniform 20-year warming trends in July of maximum and minimum temperatures are approximately 2.0°C and 1.5°C and per decade, respectively, while trends in November maxima and minima are approximately 1.5°C and 1.0°C per decade, respectively. Trends during the rest of the year are less pronounced and, with few exceptions, are non-significant (Figure 4).

In summary, the most significant monthly warming trends over the 56-year record have occurred below the alpine (D1) in spring (March-April) through increases in maximum temperatures. Further increases in maxima were evident through much of the year at stations B1 and C1. Over the last 20 years, substantial increases in maximum and minimum temperatures were uncovered in July and, less significantly, November, on the order of 1–2°C per decade across the RMFR transect. Still, the rate of warming detected over the 56 and 20-years differed not only over which months, temperatures (max, mean) and sites have warmed most, at times, it also differed in the detected scale of change. For example, over the last 56 years, the rate of max warming in July at B1 and C1, respectively, has been 0.30 and 0.60°C per decade, while over the last 20 years, the rate of max warming in July at B1 and C1, has increased by 1.9 and 1.7°C per decade, respectively (Figure 3 and 4).

### Interpolation of Climate Data Along the RMFR Transect

A comparison of PRISM’s interpolated yearly max, min and mean values with that of the RMFR’s 56-year record showed variable and often strikingly low correlations between the data sets (Table 3). At NOAA and at A1 we found relatively strong positive relationships between the interpolated yearly maximum values and the values directly recorded at the weather stations (r^2^>0.75). While correlations were also significant at the other sites, the relationships were much weaker (r^2^<0.30). The PRISM data were weakly correlated with the minimum yearly values at all sites (r^2^<0.20) and the linear relationships were not significant at B1 and D1. Mean monthly temperature values, derived from averaging PRISM’s interpolated maximum and minimum temperatures, were significantly positively correlated with the mean RMFR temperature values at each site, with stronger relationships at NOAA, A1 and B1 (r^2^ range = 0.48–0.57), and weaker correlations at C1 and D1 (r^2^<0.15) (Table 3).

**Table 3:** The relationship between PRISM’s interpolated yearly temperature values and those of the RMFR transect weather stations over the last 56-years.

	NOAA	A1	B1	C1	D1
**Max Temp**	0.82***	0.77***	0.28**	0.10*	0.13**
**Min Temp**	0.10*	0.18*	0.001	0.11*	0.04
**Mean Temp**	0.48***	0.57***	0.55***	0.15**	0.12*

Significance of r^2^ values,

*** p<0.001,

** p<0.01,

* p<0.05.

The relatively low correlation between the interpolated 56-year climate record along the RMFR transect and the actual weather station data, especially at the higher alpine sites, led to differences in the degree, and at times even direction, of perceived climate change over the same time period as recorded from the weather stations (Table 4). For example, the maximum temperatures of C1 based on station data shows a strong increase in yearly temperatures over the 56-year record while the PRISM data predicts a clear decrease in temperature during this same time period. For the fifteen measurements of slopes (three temperature measurements each at five sites), eight of them show PRISM and station data trending in different directions.

**Table 4:** Direction and rates of change in max, min and mean temperature (±SD) over the past 56 years projected by the RMFR climate stations and interpolated by PRISM.

		NOAA	A1	B1	C1	D1
**Max Temp**	RMFR	0.009±0.007 A	0.015±0.007 A	0.042±0.007 A	0.045±0.009 A	0.007±0.010 A
	PRISM	0.027±0.007 A	0.003±0.007 A	−0.002±0.007 B	−0.020±0.006 B	−0.033±0.006 B
**Min Temp**	RMFR	−0.025±0.005 A	0.001±0.005 A	−0.001±0.008 A	−0.003±0.007 A	−0.005±0.009 A
	PRISM	0.020±0.005 B	0.030±0.009 B	0.029±0.007 B	−0.011±0.006 A	−0.018±0.007 A
**Mean Temp**	RMFR	−0.008±0.006 A	0.007±0.006 A	0.017±0.005 A	0.021±0.008 A	0.001±0.009 A
	PRISM	0.024±0.006 B	0.017±0.007 A	0.014±0.006 A	−0.016±0.005 B	−0.025±0.005 A

Differences between actual (RMFR) and interpolated (PRISM) slopes for a given temperature within a site is denoted by different letters.

### Growing Season Length and Growing Degree-Day Accumulation

Analysis of the 56-year record reveals a significant shortening of the growing season in the subalpine (C1) of approximately four days per decade (Table 5), and this trend is accompanied by a significant advancement of the first autumn frost. At other elevations, there is a non-significant but consistent tendency toward a shortening of the growing season and an advancement of first autumn frost across the entire transect. Analysis of the 20-year record reveals neither trends nor consistent tendencies in growing season length, first autumn frost, or last spring frost (Table 6).

**Table 5:** Fifty-six-year trends (1953–2008) in growing season length, first autumn frost, last spring frost, and growing degree days.

Site	Growing season length(days/decade)	Last spring frost(days/decade)	First Autumn Frost(days/decade)	Growing degree days(GDD/decade)
**NOAA**	−0.68	0.41	−0.28	−5.83
**A1**	−0.44	−0.80	−1.24	13.31
**B1**	−0.90	−0.20	−1.11	35.21**
**C1**	−4.37**	1.51	−2.86**	33.49**
**D1**	−0.62	0.31	−0.31	15.38**

Rates of change (slopes) were derived from linear regression.

** p<0.01,

* p<0.05.

**Table 6:** Twenty-year trends (1998–2008) in growing season length, first autumn frost, last spring frost, and growing degree days.

Site	Growing season length(days/decade)	Last spring frost(days/decade)	First Autumn Frost(days/decade)	Growing degree days(GDD/decade)
**NOAA**	−1.87	6.62	4.74	114.5*
**A1**	0.53	−0.38	0.15	110.0**
**B1**	−0.56	2.43	1.88	88.4*
**C1**	−2.51	−0.23	−2.74	40.6
**D1**	1.23	−1.07	0.16	48.7

Rates of change (slopes) were derived from linear regression.

** p<0.01,

* p<0.05.

Analysis of the 56-year record revealed significant increases in GDDs at sites B1 through D1 (Table 5). These increases were 35, 33, and 15 GDD per decade for B1, C1, and D1, respectively. Over the 20-year record, the largest and most significant increases in GDDs occurred at lower elevations: NOAA, A1, and B1 produced increases of 114, 110, and 88 GDDs per decade, respectively (Table 6). Non-significant trends at C1 and D1 over this period showed increases of approximately 45 GDDs per decade at both sites.

## Discussion

The results of these analyses show several general patterns: 1) All five study sites demonstrated significant increases in either mean or maximum temperatures (or both), although not all sites showed significant increases over both the 20-year and 56-year records; 2) Minimum temperatures generally show less dramatic changes, but subalpine and alpine minima have clearly warmed over the last 20 years while minima at NOAA have cooled significantly over the 56-year record; 3) Analysis of monthly patterns over the 56 and 20-year records show that most warming along the RMFR transect has occurred in the spring and summer; 4) Growing season length and timing have changed little while annual GDDs have increased at all elevations, most dramatically below the subalpine over the past 20 years; 5) Trends based on station data and PRISM data often show only weak correlations and may differ in direction of change. Below we further examine these general patterns and provide a comparison with other regional and intra-continental studies.

Average yearly mean temperature trends between 1953 and 2008 indicate that significant warming along the RMFR transect is confined to the upper montane (B1, 2591 m) and subalpine (C1, 3048 m) sites and that the mean temperatures in the alpine tundra (D1, 3749 m) have remained unchanged. A similar pattern of warming at middle elevations, paired with a lack of warming in the alpine, was previously documented in the Rocky Mountain Front Range in a study of the period 1953–1997 [24]. However, this previous study documented an absolute cooling in the alpine, which was not recovered here after the incorporation of an additional 11 years of climate data. The loss of an alpine cooling signal with the addition of data from the period 1998 to 2008 suggests the alpine has been warming in recent years. Indeed, an analysis of average yearly mean temperatures over the last 20 years indicates a significant warming trend in the alpine that is not evident through analysis of 56-year trends (Figure 3).

In contrast to the elevation-dependent trends uncovered over the last 56 years, trends in average yearly mean temperatures over the last 20 years (1989–2008) show a near-uniform (albeit generally non-significant) warming of approximately 0.4°C per decade across all elevations of the RMFR transect, suggesting that warming over the last 20 years has not been biased toward any particular elevation in the Front Range, and that contemporary and future warming might be less dependent on elevation than the 56-year record suggests.

When trends in average yearly maxima and minima are examined independently, more complicated patterns emerge. A clear maximum-minimum temperature trend decoupling was uncovered over both the 20 and the 56-year analyses. On a 56-year timescale, significant warming signals are present in average yearly maxima but not in average yearly minima. These findings suggest the RMFR transect may not reflect global [43]–[45] or even regional [46] trends which attribute detected warming patterns to increasing minimum temperatures rather than (or more than) increasing maximum temperatures.

Warming patterns along the RMFR transect do not follow the general Northern Hemisphere trend of increasing winter temperatures over the last half of the twentieth century [47], although increasing spring temperatures have been previously documented in Western North America [48]. Still, multiple studies have noted decreasing summer temperatures in Northeastern Colorado [23], [49], more specifically during July [49]. While temperatures at our lowest elevation site (NOAA) generally conform to this regional cooling trend, higher elevations demonstrate significant summer warming, especially in July. This finding suggests that local climate forcing factors along the Rocky Mountain Front Range transect may override global, or even regional, climate trends.

A significant decrease in average yearly minimum temperature was uncovered at the NOAA site (1672 m) over the 56-year record. The presence of a regional cooling signal along the Front Range has been previously documented and largely attributed to changes in regional and local land-use practices over the past century, the most significant of which have occurred in the past 50 years [23], [50]. Significant cooling trends were uncovered at NOAA between May and October, consistent with suggestions that expansion of irrigated agriculture contributes to regional cooling trends in the summer and early autumn [51]. By virtue of atmospheric circulation, cooling trends associated with irrigated agriculture often become evident in adjacent regions [23], although such a cooling signal was not apparent at any of the weather stations above NOAA. In turn, the 50-year min temperature cooling at NOAA during the summer and autumn months disappears when only the 20-year record is considered. The loss of a cooling min temperature across these months suggests that other factors such as increased urbanization and/or a general increase in low land temperatures (as suggested by higher summer and autumn 20-year max temperatures) may be diminishing the cooling influence of irrigation.

Finally, the role that snow accumulation and the timing of snowmelt have played in regards to changes in seasonal temperatures during the last 20 years is not clear because within the Colorado Rockies there has been both a recorded decline in the October to April precipitation (when snow fall accumulates) and an increase in the corresponding monthly temperatures [52] and because relative changes in these variables appear to be both elevation and latitude specific [5], [52]. In turn, over the larger 50-year temporal scale changes in the snow-water equivalent (a measure of the available water held as snow) during the early season declined from 1950 to 1997 and these declines have been attributed to warming temperatures and earlier snowmelt and not to changes in precipitation [5], [53].

Future studies should explore the relationship between snow fall and snowmelt records and warming pattern at NOAA, C1 and D1 (sites where such records are available) to determine how early season and even late season temperatures and precipitation patterns are related and how changes in synoptic conditions may influence these patterns.

### Interpolation of Climate Data Along the RMFR Transect

Spatial climate data sets are in great demand by researchers and policy makers interested in linking geographic information system data to a variety of models and decision making tools [27], [54]. The goal of our comparison of the 56-year climate record of the RMFR transect and PRISM data over the same period was not to provide a peer review of PRISM, but instead to determine the value of long-term data sets in light of one of the most current, high quality and commonly used interpolation models. Overall, the comparisons between the interpolated and measured RMFR weather data showed variable and often low correlations between the data sets.

As well, the estimated rates of climate change along the RMFR transect over the last 56 years often differed between the two data sets in measured rates of change, direction of change and whether the rate of change has been significant (Table 4). Of importance here is the finding that from A1 to D1, PRISM (unlike the data from the RMFR transect) suggests that over the last 56-years, the max and average temperatures decline more and more with elevation. This pattern may no doubt influences the perception that mountain systems may be more buffered from climate change than lowland areas [5], [52], when in fact the long-term record for the area suggests that mid-elevations are warming more than the lowest and highest elevations.

Despite great statistical and computational advances, interpolating high resolution climate data within mountain systems can be difficult [27]. These difficulties may be the result of many factors that affect the climate at smaller scales (complex topology, temperature inversions, rain shadows, etc.) or may result from a lack of available weather stations in both space and time [28]. The comparisons shown here show the great value of carefully deployed long-term weather stations, such as provided by the RMFR transect, in understanding climate change patterns.

### Growing Season Length and Growing Degree-Day Accumulation

Our study did not demonstrate a consistent change in growing season length in the Rocky Mountain Front Range. This finding is in contrast to most studies, including those focused on Western North America, that consistently report an increase in growing season length over the latter part of the 20^th^ century [34] which has been attributed to an earlier onset of spring [34], [55]–[60]. However, discrepancies in the form of warming temperatures and a lack of change [59] or a shortening [46] of the growing season have been previously documented for the Rocky Mountain region. A potential explanation for the discrepancy in our study is that growing season boundaries are defined by minimum temperatures, which have remained relatively unchanged in the Front Range over the last 56 years and stand in contrast to warming maximum temperatures.

The past 56 years have seen significant increases in GDD accumulation at and above the upper montane forest (B1). The increase in GDDs at these elevations over the last 56 years reflects an increase in spring and summer maximum temperatures. Although the lack of significant trends in GDDs over the 56-year record at elevations below the upper montane forest (i.e. at NOAA and A1) is in general agreement with trends found in the Western U.S. [59], analysis of the 20-year record uncovered significant and substantial increases in degree-day accumulation toward the lower end of the RMFR transect. Trends at NOAA, A1, and B1 have significant positive slopes of roughly 100 GDD per decade over the last 20 years.

In closing, our study suggests that calculated temperature trends along the RMFR are dependent on both a) elevation and b) the temporal scale of analysis. That perceived climate change patterns in mountainous systems may depend on the temporal scale of available climate data suggests that the lack of temporal standardization and availability of long term climate records may influence our ability to understand and compare general patterns and rates of climate changes across mountain systems. While the temperature records of the last two decades suggest an elevated rate of warming across the RMFR transect, it is important to note that a continuation of this warming may depend on larger scale oscillation patterns, such as the El Niño -Southern Oscillation and the Pacific Decadal Oscillation, which have been shown to influence relative warming and cooling periods across the Rocky Mountains [61]. Continued monitoring and analysis of the data from this RMFR transect are crucial for understanding the long term trends and further transect studies at other sites will provide a clearer view of how climate change has and will continue to impact montane regions of the world.

## Supporting Information

Appendix S1 Climate data for the RMFR transect weather stations (1953–2008) with flagged corrections and additions.
(ODS)
